# Aging and Pathological Conditions Similarity Revealed by Meta-Analysis of Metabolomics Studies Suggests the Existence of the Health and Age-Related Metapathway

**DOI:** 10.3390/metabo14110593

**Published:** 2024-11-04

**Authors:** Petr G. Lokhov, Elena E. Balashova, Dmitry L. Maslov, Oxana P. Trifonova, Alexander I. Archakov

**Affiliations:** Institute of Biomedical Chemistry, Pogodinskaya St. 10, Moscow 119121, Russia; balashlen@mail.ru (E.E.B.); dlmaslov@mail.ru (D.L.M.); oxana.trifonova@ibmc.msk.ru (O.P.T.);

**Keywords:** metabolomics, aging, pathological conditions

## Abstract

**Background**: The incidence of many diseases increases with age and leads to multimorbidity, characterized by the presence of multiple diseases in old age. This phenomenon is closely related to systemic metabolic changes; the most suitable way to study it is through metabolomics. The use of accumulated metabolomic data to characterize this phenomenon at the system level may provide additional insight into the nature and strength of aging–disease relationships. **Methods**: For this purpose, metabolic changes associated with human aging and metabolic alterations under different pathological conditions were compared. To do this, the published results of metabolomic studies on human aging were compared with data on metabolite alterations collected in the human metabolome database through metabolite set enrichment analysis (MSEA) and combinatorial analysis. **Results**: It was found that human aging and pathological conditions involve the set of the same metabolic pathways with a probability of 99.96%. These data show the high identity of the aging process and the development of diseases at the metabolic level and allow to identify the set of metabolic pathways reflecting age-related changes closely associated with health. Based on these pathways, a metapathway was compiled, changes in which are simultaneously associated with health and age. **Conclusions**: The knowledge about the strength of the convergence of aging and pathological conditions has been supplemented by the rigor evidence at the metabolome level, which also made it possible to outline the age and health-relevant place in the human metabolism.

## 1. Introduction

Simultaneously with a dropping birth rate, the lifespan of people worldwide has been continuously rising in recent decades [1]. Population aging is now a widespread phenomenon and one of the most significant social changes in the twenty-first century, posing economic and social challenges, particularly in the area of health care, given that aging is often accompanied by cardiovascular, respiratory, and mental diseases, arthritis, diabetes, and disability [2,3]. To find ways to reduce aging symptoms, researchers continue to explore the molecular mechanisms of aging [4,5].

Aging causes the body to undergo numerous changes at all organizational levels, from genome to metabolome. However, there is still much to be understood about the molecular basis of aging. Over the past 30 years, gerontological research has made significant strides in understanding how genes regulate aging [6,7]. Genomic studies place a lot of emphasis on the variables that influence lifespan and successful aging, defined as the absence of chronic diseases and the capacity to act well at physiological and psychological levels [8,9,10,11]. However, the intricate combination of multiple factors (ranging from hereditary to numerous environmental ones) determines a lifespan [12]. Transcriptomics, proteomics, and metabolomics are examples of fundamental postgenomic omics sciences that can reveal further details regarding alterations in the organism at “post-genome” molecular levels [13,14,15]. Metabolomics holds a unique position in scientific research since the metabolome serves as the culmination point for biological events that emerge from complex interactions between genes, proteins, biochemical processes, and environmental factors [16,17,18]. Metabolites, low-molecular compounds that are substrates, intermediates, and products of metabolic events taking place in the body, form metabolomes [4,19]. As a result, the metabolome analysis can offer details on the body’s current metabolic status in relation to physiological and pathological processes [20,21] as well as reveal the metabolic signatures of organism aging [22,23,24].

**Figure 1:**
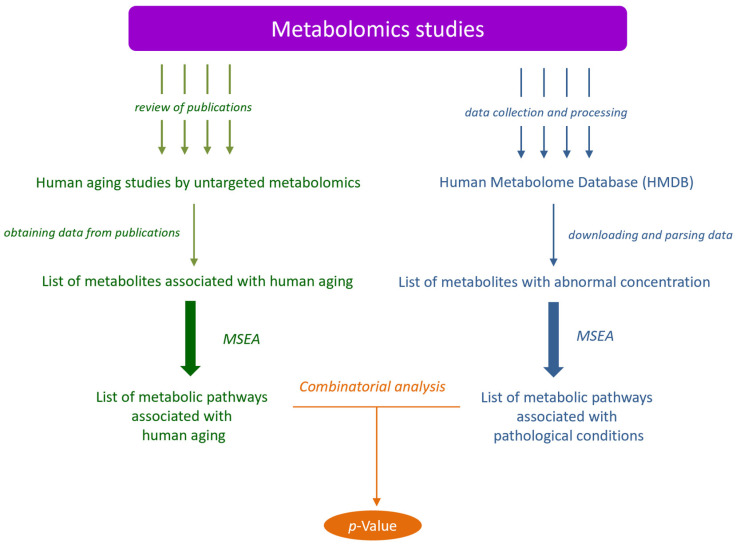
Workflow for comparison of metabolomic data collected on human aging and pathological conditions. *p*-value indicates a probability that the lists of human aging-related pathways and pathways associated with pathological conditions are the same. MSEA, metabolite set enrichment analysis.

For the global metabolome overview, untargeted metabolomics approaches measuring bid sets of metabolites are ideally suitable [22,25,26,27,28,29,30]. In a previous study, a comparative analysis of untargeted metabolomics aging studies in various animal models (from C. elegans to mammals) and humans was conducted using metabolite set enrichment analysis (MSEA), which showed the identity of aging among them from a metabolomics point of view [31]. This result stimulated further study of the aging process using the same approach. The purpose of this work is to compare metabolic changes associated with human aging with metabolic alterations defined in pathological conditions. To do this, the published results of untargeted metabolomic studies on human aging were compared with data on human metabolite alterations at different pathological conditions collected in the human metabolome database (Figure 1).

## 2. Materials and Methods

### 2.1. Metabolite Datasets

Studies focusing on untargeted metabolomic analysis of the blood metabolome during aging [32,33,34,35,36,37] have been previously reviewed [31]. The aging-associated metabolites in humans identified in these studies were used as metabolite dataset 1.

Metabolites associated with human pathological conditions were obtained from the Human Metabolome Database (HMDB; https://hmdb.ca; accessed on 1 September 2022) and used as metabolite dataset 2. For this, the database was downloaded in XML format from the HMDB website, parsed in MATLAB program (version R2019a; MathWorks, Natick, MA, USA), and dataset 2 with metabolites at abnormal concentrations and associated pathological conditions was compiled.

### 2.2. Metabolite Set Enrichment Analysis

MSEA [38] was applied using MetaboAnalyst 5.0 software (www.metaboanalyst.ca; accessed on 1 September 2023) [39] with the following options: module, ‘pathway analysis’; input type, ‘compound names’ or ‘KEGG ID’; visualization method, ‘scatter plot (testing significant features)’; enrichment method, ‘hypergeometric test’; topology analysis, ‘relative-betweenness centrality’; reference metabolome, ‘use all compounds in the selected pathway library’; pathway library, ‘homo sapiens (KEGG)’ (80 pathways; KEGG pathway info were obtained in October 2019). The enrichment analysis (type over-representation analysis (ORA)) implemented using the hypergeometric test evaluated whether a particular metabolite set was represented more than expected by chance within the given compound list. One-tailed *p*-values were provided by ORA after adjusting for multiple testing.

### 2.3. Combinatorial Analysis

To assess whether the presence of the same metabolic pathways associated with human aging and pathological conditions is statistically significant, combinatorial analysis was used. Namely, the combinatorial problem was solved: the calculation of the probability that a defined set of metabolic pathways associated with human aging could appear among metabolic pathways associated with pathological conditions.

### 2.4. Formation and Analysis of Health and Aging-Related Metapathway

Pathways enriched both in aging and in pathological conditions were used to compile health and aging-related metapathway. This metapathway was used to apply enrichment analysis with the same parameters as described in Section 2.2. For enrichment analysis, the option of MetaboAnalyst 6 for uploading custom metabolite pathways (www.metaboanalyst.ca; accessed on 15 August 2024) was used to add metapathway to default KEGG pathway-based 80 metabolite sets (KEGG pathway info was obtained in December 2023).

Projection of metabolites, included in metapathway, onto KEGG pathways was conducted by the network analysis module of the MetaboAnalyst 6 (option ‘KEGG global metabolic network’; accessed on 8 September 2024). The metabolite–metabolite interaction network for metabolites included in the metapathway was built using the same module (option ‘metabolite–metabolite interaction network’; layout Fruchterman–Reingold). According to the module description, the chemical–chemical associations for the metabolite network were extracted from STITCH [40], so that only highly confident interactions are used.

## 3. Results

### 3.1. Metabolic Pathways Associated with Human Aging and Pathological Conditions

In a previous study [31], untargeted metabolomic studies of blood have been reviewed in order to observe human metabolome changes during aging [32,33,34,35,36,37]. The obtained list of human aging-associated metabolites, including study reference, sample type, age of involved subjects, and metabolite detection method, is presented in Supplementary Table S1. This set of aging-associated metabolites was used as metabolite dataset 1 in this study.

In accordance with the goal of this study, which is to compare metabolic changes linked to human aging with metabolic variations under various pathological conditions, dataset 2 was compiled from the HMDB using data on metabolites at abnormal concentrations under 326 pathological conditions. The resulting metabolite dataset 2 is presented in Supplementary Table S2 (list of pathological conditions with corresponding metabolite identifiers).

**Figure 2:**
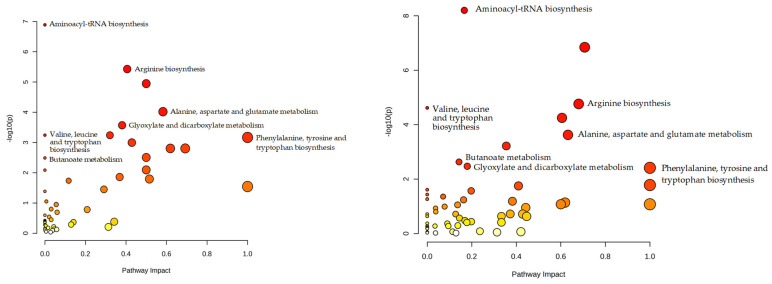
Results of metabolite set enrichment analysis (MSEA). (**a**) Metabolic pathways potentially involved in human aging. The graph was generated by projecting metabolites associated with human aging (metabolite dataset 1) onto metabolic pathways using MSEA. (**b**) Metabolic pathways potentially involved in human pathological processes. The graph was generated by projecting metabolites for which abnormal concentrations are described in the Human Metabolome Database (metabolite dataset 2) onto human metabolic pathways using MSEA. The names of metabolic pathways concurrently associated with human aging and pathological conditions (i.e., enriched with projected metabolites with an adjusted *p*-value < 0.05) are shown. The *p*-value, determined by the pathway enrichment analysis, evaluates whether a measured set of metabolites is represented in the pathway more than expected by chance within a given list of metabolites. Pathway impact values are from the pathway topology analysis.

Both metabolite datasets were subjected to MSEA. Figure 2 and Table 1 show metabolic pathways, which are considered to be involved in human aging and pathological conditions based on projecting the metabolites in dataset 1 and dataset 2 onto human metabolic pathways by MSEA.

Based on the MSEA, it may be concluded that the untargeted metabolomic data collected to date on human aging suggests that the 15 metabolic pathways are statistically significantly involved in the aging process. The information gathered from metabolomic studies on various metabolite alterations and stored in the HMDB thus far indicates the statistically significant involvement of 11 metabolic pathways. Among them, seven metabolic pathways were found from the MSEA of metabolites from metabolomic studies on human aging (see checkmarked pathways in Table 1).

### 3.2. Combinatorial Analysis Result

To confirm that the MSEA results for human aging-related metabolites and pathological conditions describe the same biological phenomenon, combinatorial analysis was used. The probability (p) that seven human aging-related pathways were non-randomly found among pathological condition-related pathways was calculated.

An elementary event—there are 15 human aging-associated metabolic pathways. An evaluated event: out of 11 pathological condition-related metabolic pathways, 7 are human aging-related (presuming that the processes of aging and pathological conditions reflected in metabolic pathways are not independent). Considering that there are only 80 human metabolic pathways, by choosing 11 metabolic pathways out of 80, a sample set can be obtained by combination:

$${n=C}_{80}^{11}=\frac{80!}{11!\left(80-11\right)!}$$

Non-aging-related pathways could appear among pathological condition-related pathways by selecting 4 pathways out of 65 pathways, which gives the total number of such samples:

$${m1=C}_{65}^{4}=\frac{65!}{4!\left(65-4\right)!}$$

Similarly, the number of possible groups of 7 aging-related pathways is determined by a combination of 7 out of 15:

$${m2=C}_{15}^{7}=\frac{15!}{7!\left(15-7\right)!}$$

The aging-related and non-aging-related pathways fall into the set of pathological condition-related pathways independently of each other; therefore, to calculate the number of elementary events favorable to an evaluated event, the multiplication rule (“and” rule) of combinatorics was used. So, the total number of favorable elementary events:

m=m1×m2

The probability of the evaluated event was determined by the formula:

$$p=1-\frac{m}{n}=0.9996(or\;99.96\%)$$

The resulting probability value indicates that aging-related and pathological condition-related pathways reflect or belong to the same biological phenomenon with a probability of 99.96%.

### 3.3. Health and Aging-Related Metapathway

Metabolic pathways associated with both aging and pathological conditions (Table 1), such as ‘Arginine biosynthesis’, ‘Valine, leucine, and isoleucine biosynthesis’, ‘Alanine, aspartate, and glutamate metabolism’, ‘Butanoate metabolism’, ‘Glyoxylate and dicarboxylate metabolism’, ‘Phenylalanine, tyrosine, and tryptophan biosynthesis’, except for ‘Aminoacyl-tRNA biosynthesis’, were combined into health and aging-related metapathway. The enrichment of the metapathway by both aging and pathological conditions-associated metabolites was equally high (*p*-value of 3.6 × 10^−12^ and 1.3 × 10^−10^, respectively; Table 1).

The metabolite–metabolite interaction network helps to highlight potential functional relationships between metabolites. Using data from STITCH (‘search tool for interactions of chemicals’) [40], which integrate information about interactions from metabolic pathways, binding experiments, and drug–target relationships, to construct an interaction network, it is possible to construct a full-fledged interaction network based on metabolites detected in metabolomic studies, supplementing them with interacting but not detected metabolites. The network also identifies metabolites with the highest number of functional connections (degree of nodes), which determines their importance in the network.

**Figure 3:**
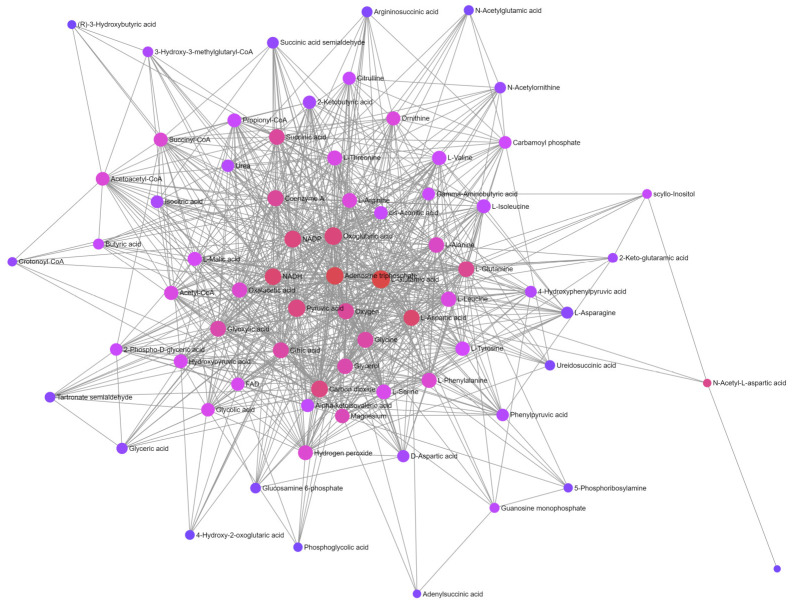
Metabolite–metabolite interaction network for metapathway metabolites. Circle size and color correspond to the degree number of the node. Degree values for central nodes (metabolites), indicated as large red circles, are presented in Table 2 (data for all metabolites are presented in Supplementary Table S3).

Such an interaction network built for metabolites of the metapathway (Figure 3) shows multiple connections between them, indicating the functional integrity of the metapathway. Metabolites with the largest number of connections (metabolites in the center of the network) are presented in Table 2.

The data in Table 2 indicate that the central role in the metapathway is assigned to metabolites related to energy metabolism. Such metabolites as ATP, NADH, NADP, carbon dioxide, oxygen, and pyruvic acid are directly related to cellular respiration and reflect the cellular energy state. Oxoglutaric acid, as a TCA intermediate, and glutamic acid, which is converted from oxoglutaric acid using NADP, are also highly relevant to energy metabolism.

## 4. Discussion

A useful tool for researching aging-related biological processes is metabolome analysis [16,25,29]. Metabolome analysis aims to measure many substances with low molecular weights simultaneously in biological samples [41,42,43]. Nuclear magnetic resonance spectroscopy [44,45] and mass spectrometry [46,47,48] provide information about large sets of metabolites in a biological sample in a single analysis. Pico- and femtomole concentration analysis of hundreds or thousands of metabolites in a sample is possible with mass spectrometers [49]. As a result, metabolomics allows gathering information on a wide range of metabolites belonging to different chemical classes and metabolic pathways.

Two main types of metabolome analysis can be identified: targeted for measuring predefined sets of metabolites and untargeted, which is related to panoramic detection of sample metabolites [28,34,50]. According to the aim and design of this study, the results of untargeted (panoramic) studies are more suitable since the results of targeted studies are limited to predefined sets of metabolites, making it difficult to draw generalized conclusions. Moreover, MSEA, being a central method in this study, distorts the results when a predefined set of metabolites is used. A review of untargeted metabolomics studies related to aging was conducted in a previous study [31], and the human aging data from that review were used in this study as dataset 1.

A wide investigation of alterations in metabolic pathways was conducted through analysis of metabolomes over the past two decades. Several databases collect results from these studies, where metabolites are annotated with information about their chemical composition, method of detection, concentration data for both normal and pathological conditions, and metabolic pathways in which they participate. HMDB is one of the most well-known databases with detailed descriptions of metabolites that have been identified and present in the human body. The data on metabolites with abnormal concentrations were extracted from this database to compile dataset 2 for comparison with dataset 1.

**Figure 4:**
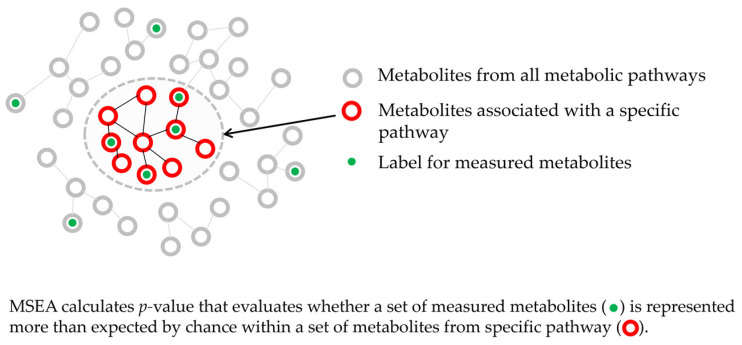
Principle of metabolite set enrichment analysis (MSEA) applied to metabolic pathways.

When considering the results of different metabolomic studies to draw generalized conclusions, it is necessary to take into account the specifics of such studies. The measurement of large sets of metabolites using different samples, sample preparation protocols, and measurement equipment leads to different sets of metabolites being measured in untargeted metabolomic studies. This makes it difficult to generalize findings from the different studies that are directly relevant to our study. A review of the most frequent metabolites in dataset 1 and dataset 2 (Supplementary Figures S1 and S2) confirms that finding a set of metabolites common to both datasets is challenging. This problem can be solved by the projection of identified metabolites from different studies onto metabolic pathways. If different metabolites found in different studies are projected onto the same metabolic pathway, this indicates their participation in identical biochemical processes, and such data can be interpreted in the same way. MSEA, which enables getting a statistical estimate of such projection, is one of the popular methods for doing this [51]. MSEA provides the *p*-value that the measured metabolites match a certain metabolite set, in particular a metabolic pathway (Figure 4). If different metabolites detected in different studies are involved in the same metabolic pathway, this suggests that they are involved in the same biochemical processes. Therefore, in this research, the projection of datasets 1 and 2 into metabolic pathways using MSEA is the most appropriate way to compare them.

Combinatorial analysis of MSEA results suggested that metabolomic data on aging and pathological conditions have a high degree of identity. Of the fifteen aging-related pathways, seven appeared among the eleven pathways associated with pathological conditions. Combinatorial analysis indicates that we observe similar processes with a probability of 99.96%. Moreover, the similarity is even more pronounced than the resulting probability conveys. For example, the presence of the steroid hormone biosynthesis pathway among pathological condition-related pathways and the absence among aging-related pathways, although the relationship between steroids and aging is well-known, may be explained. Several steroids collected from the aging-related studies were not recognized and, therefore, not processed by MetaboAnalyst due to the diversity of their structural conformations and the way their names are spelled. So, the difference in steroids between MSEA results is more a limitation of method than a real difference. With this comment in mind, the difference between pathways associated with aging and pathological conditions becomes even less defined.

**Figure 5:**
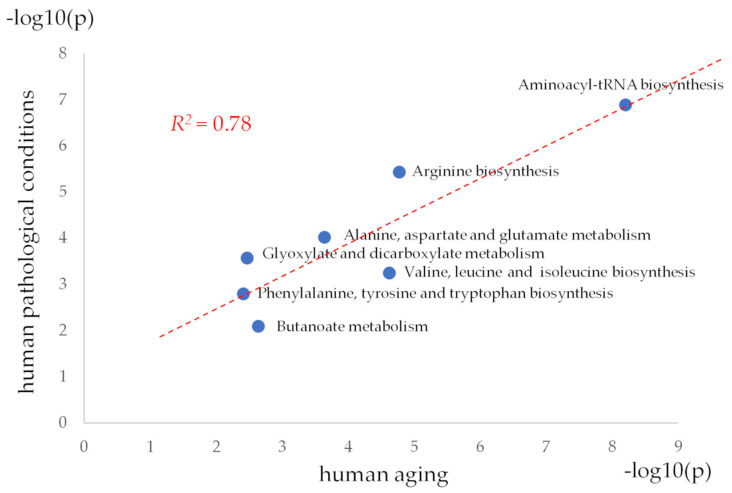
Scatter plot of human aging-associated metabolic pathways versus pathological condition-associated pathways. The *p*-value evaluates whether a measured set of metabolites is represented in the pathway more than expected by chance within a given list of metabolites (*p*-values from the pathway enrichment analysis). *R*^2^ is a coefficient of determination calculated for the linear approximation of scatter plot points.

Moreover, matched aging-related pathways and abnormal metabolite-related pathways have close ranking orders. In the first place in both cases is ‘Aminoacyl-tRNA biosynthesis.’ ‘Arginine biosynthesis’, ‘Alanine, aspartate, and glutamate metabolism’, ‘Valine, leucine, and isoleucine biosynthesis’ occupy an intermediate position. ‘Phenylalanine, tyrosine, and tryptophan biosynthesis’ and ‘Butanoate metabolism’ are towards the end of the list. This pattern is another sign in favor of the identity of MSEA results for dataset 1 and dataset 2. If a scatter plot of these metabolic pathways is built (Figure 5), then they can be linearly approximated with a coefficient of determination (*R^2^*) equal to 0.78, which objectively confirms the presence of similarity in their ranking for pathological conditions and aging.

The obtained results allow us to state that the development of pathological conditions and aging have great similarities at the metabolic level, which, in some way, allows us to assume that they are related to the same general biological process. It is quite possible to find confirmation of this statement in the scientific data accumulated to date. The metabolome is a molecular phenotype and is the collector of all biochemical events in the organism since metabolites are substrates, intermediates, and end products of biochemical reactions combined in the metabolic pathways. Therefore, it is not surprising that the aging of the organism has a systemic reflection in the metabolome. Based on the previous metabolomic studies, several major metabolic pathways, e.g., related to lipids and lipoproteins, steroid hormones, the renal system and excretion, amino acids and muscle, diet, oxidative stress, and inflammation, are involved in aging [52]. On the other hand, the morbidity and overall mortality from the most common diseases with age are so obvious that it even allows us to consider aging as a risk factor for diseases [53]. These established links between aging, metabolomes, and diseases make the existence of connections between aging and diseases quite expected. From this point of view, this work simply revealed this connection. The novelty here is that evidence of this connection has been obtained with mathematical accuracy for the ‘ome’ level, which makes it possible to make reliable conclusions at the level of the organization of living systems.

The revealed identity in the lists of the metabolic pathways altered in aging and pathological conditions provokes the outline of the set of pathways, which is essentially a metapathway (“meta-” means compiled from several pathways). This metapathway is the main collector of both age-related and pathological changes. The metapathway, formed by combining six metabolic pathways, is simultaneously highly enriched with metabolites associated with both aging and pathological conditions (Table 1). The definition of this metapathway has scientific significance because it points to the part of metabolism (amino acid metabolism, butanoate metabolism, glyoxylate metabolism, and dicarboxylate metabolism) associated with the development of pathologies and aging simultaneously.

Although the study design implies that the metabolic pathways included in the metapathway are relevant to diseases and age-related changes, the following brief review of the accumulated scientific evidence on the involvement of the key metabolites of these pathways in the aging process and disease development provides additional support for the designation of the metapathway.

*Amino Acids*

Omitting the huge amount of data on the influence of amino acids on diseases, age-related diseases, and lifespan [54], below are some facts confirming the rationale for including the four amino acid-related pathways in the metapathway.

Branched-chain amino acids (BCAAs), including valine, leucine, and isoleucine, are the most abundant essential amino acids, and diet is the main significant source of BCAAs for humans [55]. They play key roles in the regulation of many physiological processes and aging. Many studies report findings on the relationship between BCAA blood levels and age-related changes in body composition, physical function, sarcopenia, obesity, insulin and glucose metabolism, and the biology of aging itself [56,57,58,59].

Studies in large population cohorts have demonstrated a pathophysiological role for arginine metabolites in major chronic diseases in old age, particularly for vascular disease and atherosclerosis [60]. Using a longitudinal approach, phenylalanine, tyrosine, and tryptophan metabolism was shown to be altered with aging in the plasma in the primate aging model [61,62]. Elevated serum phenylalanine has been linked to telomere loss in men [63], inflammatory disease [64], and type 2 diabetes [65]. Dietary protein restriction in rats increased lifespan and decreased phenylalanine levels in liver [66].

Studies have been performed focusing on tryptophan in the following aging-related disorders: cardiovascular disease [67,68,69], chronic kidney disease [70], diabetes [71], depression [72], inflammatory bowel disease [73], and multiple sclerosis [74]. Tryptophan levels were most frequently lower in the disease state, and supplemental tryptophan most often decreased disease phenotypes. Dietary tryptophan restriction activates specific anti-aging pathways, resulting in rats’ delayed reproductive aging [75] and extended lifespan [76,77].

Tyrosine is the precursor for the neurotransmitters dopamine and norepinephrine. In a metabolomics study of human plasma, tyrosine levels increased with age [35]. Increased dietary tyrosine levels are linked with increased cognitive performance [78]. Tyrosine supplementation enhances dopaminergic neurotransmission in Parkinson’s disease patients [79]. High levels of tyrosine in the plasma increase the risk of type 2 diabetes [65].

Increased alanine consumption may be beneficial for metabolic disorders such as type 2 diabetes, as its addition to a cultured pancreatic beta cell line increases both glucose metabolism and the secretion of insulin [80]. Alanine supplementation has also been shown to stimulate the proliferation of lymphocytes [81] and thymocytes [82], which may decrease the aging-related loss of immune system function. In an animal model, dietary alanine prevents obesity [83]. Alanine levels decline with aging in mouse plasma [47] and muscle [84]. The metabolism of alanine to pyruvate may play a role in the extended longevity of nematodes [85].

Aspartate participates in a range of important cellular functions, such as the urea cycle and the malate–aspartate shuttle. Mitochondrial electron transport chain function declines with aging [86] and is required for aspartate synthesis [87]. Metals bind to aspartate to form complexes with antioxidant function [88]. Aspartate supplementation decreases reactive oxygen species (ROS), increases ATP levels [89], and decreases hepatotoxicity and oxidative stress [90]. Aspartate regulates neurotransmission [91] and may contribute to aging-related cognitive impairment by facilitating excitotoxicity [92].

Glutamate plays an essential role in learning and cognition, but at high levels, glutamate induces neuronal excitotoxicity, contributing to neuronal injury in neurodegenerative disorders, including Alzheimer’s disease [93] and amyotrophic lateral sclerosis [94]. Glutamate level declined in the human brain during young adulthood [95]. Glutamate binds free ammonia, which is especially toxic to neurons [96], and it serves as an important anaplerotic source of the anti-aging citric acid cycle intermediate alpha-ketoglutarate [97]. Higher levels of glutamate were found in the plasma of long-lived rats [98].

*Butanoate Metabolism*

Butanoate metabolism was defined as one of metabolic-age predictors [99]. Butanoate metabolism has been linked to sarcopenia, deterioration of skeletal muscle, with age [100]. Aging often leads to mitochondrial dysfunction. Butyrate enhances mitochondrial activity through serving as an additional energy source and supporting ATP production in mitochondria [101]. Additionally, butyrate participates in the upregulation of anti-inflammatory genes and downregulation of pro-inflammatory genes, leading to reduced systemic inflammation associated with aging [102]. This function is crucial in preventing age-related intestinal permeability [103]. Butyrate can modulate immune responses in the gut [104]. Dysregulation of immune responses is a hallmark of aging, and butyrate’s role in immune homeostasis is of significant interest.

Moreover, butyrate’s influence also extends to telomere shortening, a hallmark of cellular aging. Butyrate’s impact on inflammation and oxidative stress may indirectly influence telomere maintenance. Chronic inflammation and oxidative stress often accelerate telomere shortening, but by reducing these factors, butyrate may help slow down the rate of telomere erosion [105]. Additionally, butyrate’s anti-inflammatory properties can help alleviate the pro-inflammatory secretions of senescent cells, reducing the harmful effects associated with cellular senescence [106].

*Glyoxylate and Dicarboxylate Metabolism*

Glyoxylate and dicarboxylate metabolism also have a large rise in contribution to metabolome variance with age [99]. It is strictly linked with glycine, serine, and threonine metabolism, pyruvate metabolism, and ascorbate metabolism, all of which have been identified as linked to aging [99]. This pathway has already been found to be associated with many metabolic diseases (type 2 diabetes, obesity, and atherosclerosis) [107,108] and microbiome composition [109], changes in which can contribute to metabolic inflammation and major age-related metabolic disorders [110,111,112].

Thus, accumulated scientific evidence points to the relevance of the pathways comprising the metapathway to aging and disease development.

It is necessary to explain why the aminoacyl-tRNA biosynthesis pathway was not included in the metapathway. In this pathway, amino acids are linked with their cognate transfer RNAs. The metapathway also largely consists of amino acid-related pathways. Therefore, double accounting of many amino acids would violate the statistical model of MSEA. The feasibility of including this pathway in the metapathway after leveling the double hit of amino acids remains to be studied.

As it was explained earlier, several steroids from the aging-related studies were not recognized by MetaboAnalyst, which may have prevented the steroid hormone biosynthesis pathway from being included in the metapathway. However, the association of steroids with aging is well established, and this pathway ranks first among metabolic pathways in correlation and prediction of chronological age [113]. Thus, there is a reason to investigate the rationale for including this metabolic pathway in the metapathway in the future.

The next remark concerns the fact that metabolic pathways have a holistic structure. The integrity of the metapathway is also present. The pathways that comprise the metapathway are locally located in the global pathway network (Supplementary Figure S3) and are connected, except for the phenylalanine, tyrosine, and tryptophan biosynthesis pathways. The metapathway was defined from the experimental data, where gaps are not uncommon. However, it is conceivable that this gap could be filled by the glycine, serine, and threonine metabolism or the TCA cycle. The first one was only associated with pathological conditions, but the relationship of related amino acids with aging is well known from the literature [54]. The TCA cycle presented only in age-related pathways is the central metabolic pathway and may be quite suitable for obtaining metapathway integrity.

The construction of a metabolite–metabolite interaction network confirmed the high functional integrity of the metapathway (Figure 3, Table 2), indicating the central role of energy metabolism in it. This is close to the mitochondrial theory of aging, where the energy metabolism is disrupted due to mitochondrial dysfunction. There is room for theorizing here. Since the metapathway is a common collector of metabolic changes during aging and various pathologies, with energy metabolism playing a central role in it, aging associated with disturbances in energy metabolism leads to deterioration in health. Conversely, healthy aging is characterized by the absence of significant changes, or only moderate changes, in energy metabolism.

Among the prospects for practical application, the use of the metapathway in metabolomic studies of aging and age-related diseases stands out. In metabolomics, enrichment analysis is extremely common. MetaboAnalyst, which was used in this work to perform enrichment analysis, has been used by >500,000 researchers (https://www.metaboanalyst.ca/docs/UserStats.xhtml; accessed on September 4, 2024). It allows to combine pathways and perform MSEA to return a *p*-value for the enrichment of the metapathway by altered metabolites. Such *p*-value is closely related to metapathway alteration and can be used in the study of human health in association with aging. The measure of metapathway alteration is also a potential way to create health-related metabolic clocks and can be useful in the study of healthy aging.

## 5. Conclusions

It was found that aging and pathological conditions at the metabolic level are part of the same processes, with a probability of 99.96%. This fact gives a new perspective on the positioning of aging and disease development as different ways of looking at the same phenomenon. The obtained high probability also provided the basis for identifying a metapathway that is simultaneously related to both aging and disease development, which is of fundamental importance. Based on metapathway composition, it was hypothesized that healthy aging can be considered as aging without alteration of energy metabolism. The practical application of the metapathway has enormous potential. Analysis of metapathway enrichment by the MSEA, which is widely used in metabolomics, may provide a metric of age and health-related changes in the organism, which is essentially the basis for developing health-related metabolic clocks.

## Figures and Tables

**Table 1:** Results of MSEA obtained by projecting metabolites associated with human aging and with abnormal concentrations in various pathological conditions onto human metabolic pathways.

#	Pathway Name ^1^		Match Status (Hits/Total) ^2^	*p*-Value	FDR ^3^
For human aging					
1	Aminoacyl-tRNA biosynthesis		14/48	1.3 × 10^−7^	1.1 × 10^−5^
2	Arginine biosynthesis		7/14	3.7 × 10^−6^	1.6 × 10^−4^
3	Histidine metabolism		7/16	1.1 × 10^−5^	3.2 × 10^−4^
4	Alanine, aspartate, and glutamate metabolism		8/28	9.6 × 10^−5^	0.0020
5	Glyoxylate and dicarboxylate metabolism		8/32	2.7 × 10^−4^	0.0045
6	Valine, leucine, and isoleucine biosynthesis		4/8	5.7 × 10^−4^	0.0069
7	Citrate cycle (TCA cycle)		6/20	5.8 × 10^−4^	0.0069
8	Phenylalanine, tyrosine, and tryptophan biosynthesis		3/4	6.8 × 10^−4^	0.0071
9	Nicotinate and nicotinamide metabolism		5/15	0.0010	0.0094
19	Phenylalanine metabolism		4/10	0.0016	0.0120
11	Caffeine metabolism		4/10	0.0016	0.0120
12	D-glutamine and D-glutamate metabolism		3/6	0.0031	0.0209
13	Biosynthesis of unsaturated fatty acids		7/36	0.0032	0.0209
14	Ascorbate and aldarate metabolism		3/8	0.0080	0.0458
15	Butanoate metabolism		4/15	0.0082	0.0458
	**Metapathway** (6 united pathways)		**23/76**	**3.6 × 10^−12^**	**2.5 × 10^−10^**
For abnormal concentrations in various pathological conditions					
1	Aminoacyl-tRNA biosynthesis	✓	20/48	6.3 × 10^−9^	5.3 × 10^−7^
2	Glycine, serine and threonine metabolism		15/33	1.4 × 10^−7^	6.0 × 10^−6^
3	Arginine biosynthesis	✓	8/14	1.7 × 10^−5^	4.8 × 10^−4^
4	Valine, leucine, and isoleucine biosynthesis	✓	6/8	2.4 × 10^−5^	5.1 × 10^−4^
4	Steroid hormone biosynthesis		21/85	5.6 × 10^−5^	9.5 × 10^−4^
6	Alanine, aspartate and glutamate metabolism	✓	10/28	2.3 × 10^−4^	0.0033
7	Tyrosine metabolism		12/42	6.1 × 10^−4^	0.0073
8	Butanoate metabolism	✓	6/15	0.0023	0.0246
9	Glyoxylate and dicarboxylate metabolism	✓	9/32	0.0034	0.0290
10	Arginine and proline metabolism		10/38	0.0035	0.0290
11	Phenylalanine, tyrosine, and tryptophan biosynthesis	✓	3/4	0.0039	0.0294
	**Metapathway** (6 united pathways)		**29/76**	**1.3 × 10^−10^**	**1.1 × 10^−8^**

^1^ Only metabolic pathways with FDR < 0.05 are presented. ^2^ ‘Hits’ is the matched number of metabolites from the datasets. ‘Total’ is the total number of compounds in the pathway. ^3^ *p*-value adjusted using False Discovery Rate (FDR). A checkmark (✓) indicates pathological condition-related pathways, which are also human aging-related.

**Table 2:** Metabolite–metabolite interaction network parameters for central nodes (metabolites).

Metabolite Name	Degree	Betweenness
L-Glutamic acid	57	234.89
Oxoglutaric acid	53	88.12
Adenosine triphosphate	51	179.78
Pyruvic acid	50	82.26
NADP	46	82.82
Carbon dioxide	46	80.72
NADH	44	108.28
Oxygen	40	52.65

## Data Availability

The data are presented in Supplementary Materials.

## Supplementary Materials

The following supporting information can be downloaded at: https://www.mdpi.com/article/10.3390/metabo14110593/s1, Figure S1: Frequency of occurrence for metabolites in untargeted metabolomics studies related to aging; Figure S2: Frequency of detection of abnormal metabolite concentration in different human body conditions; Figure S3: Projection of metapathway metabolites on the KEGG global pathway network; Table S1: List of untargeted metabolomics studies on aging and identified aging-associated metabolites; Table S2: List of metabolites with abnormal concentrations and associated conditions; Table S3: Metabolite–metabolite interaction network parameters for nodes (metabolites).
