# Supplementary material for: Aging and Pathological Conditions Similarity Revealed by Meta-Analysis of Metabolomics Studies Suggests the Existence of the Health and Age-Related Metapathway
Source: Metabolites. 2024 Nov 4;14(11):593. doi: 10.3390/metabo14110593 (PMC11597009; doi:10.3390/metabo14110593)
Supplement: Supplementary file 1 [file metabolites-14-00593-s001.zip › Supplementary Figures S1, S2, S3.pdf]

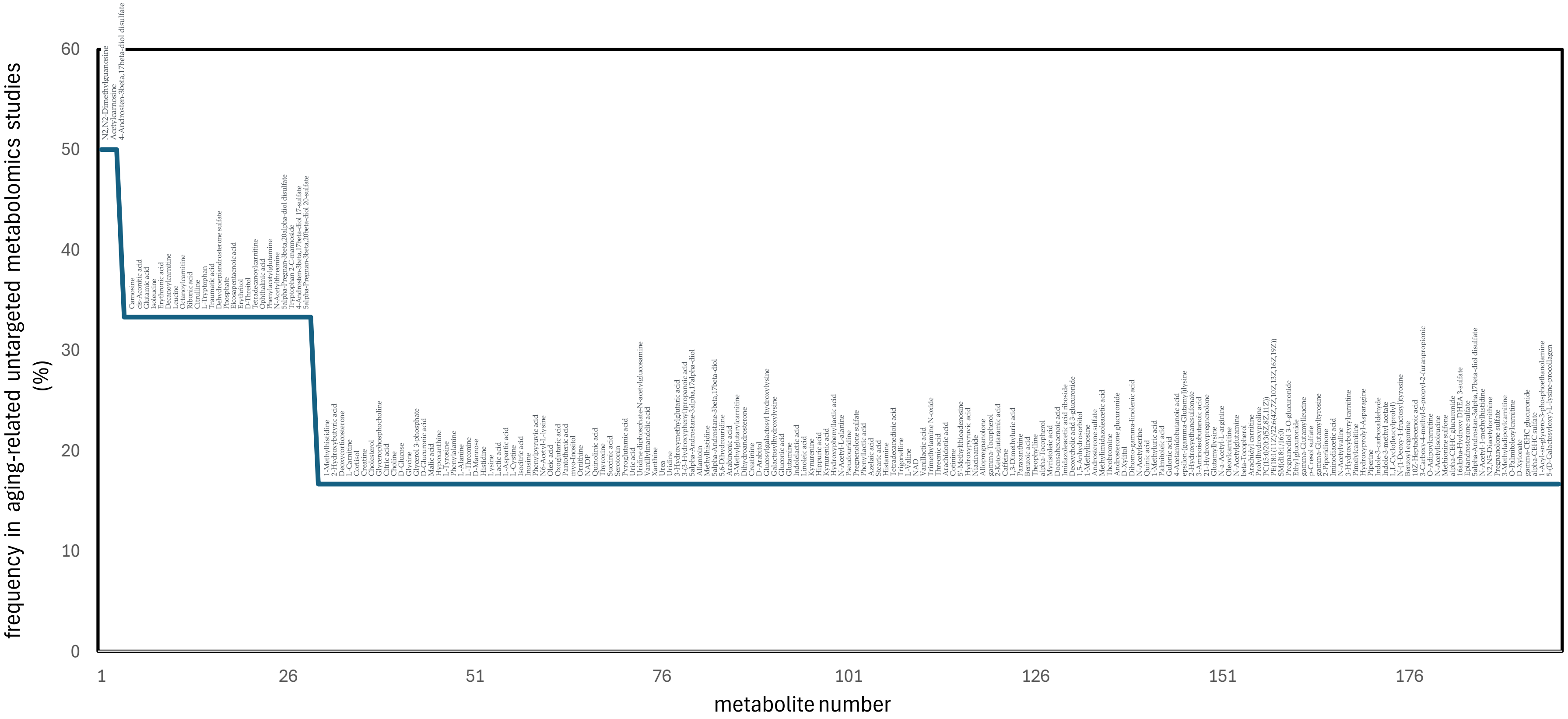

Figure S1. Frequency of occurrence for metabolites in untargeted metabolomics studies related to aging. Data were obtained from 6 scientific studies (see Table S1).

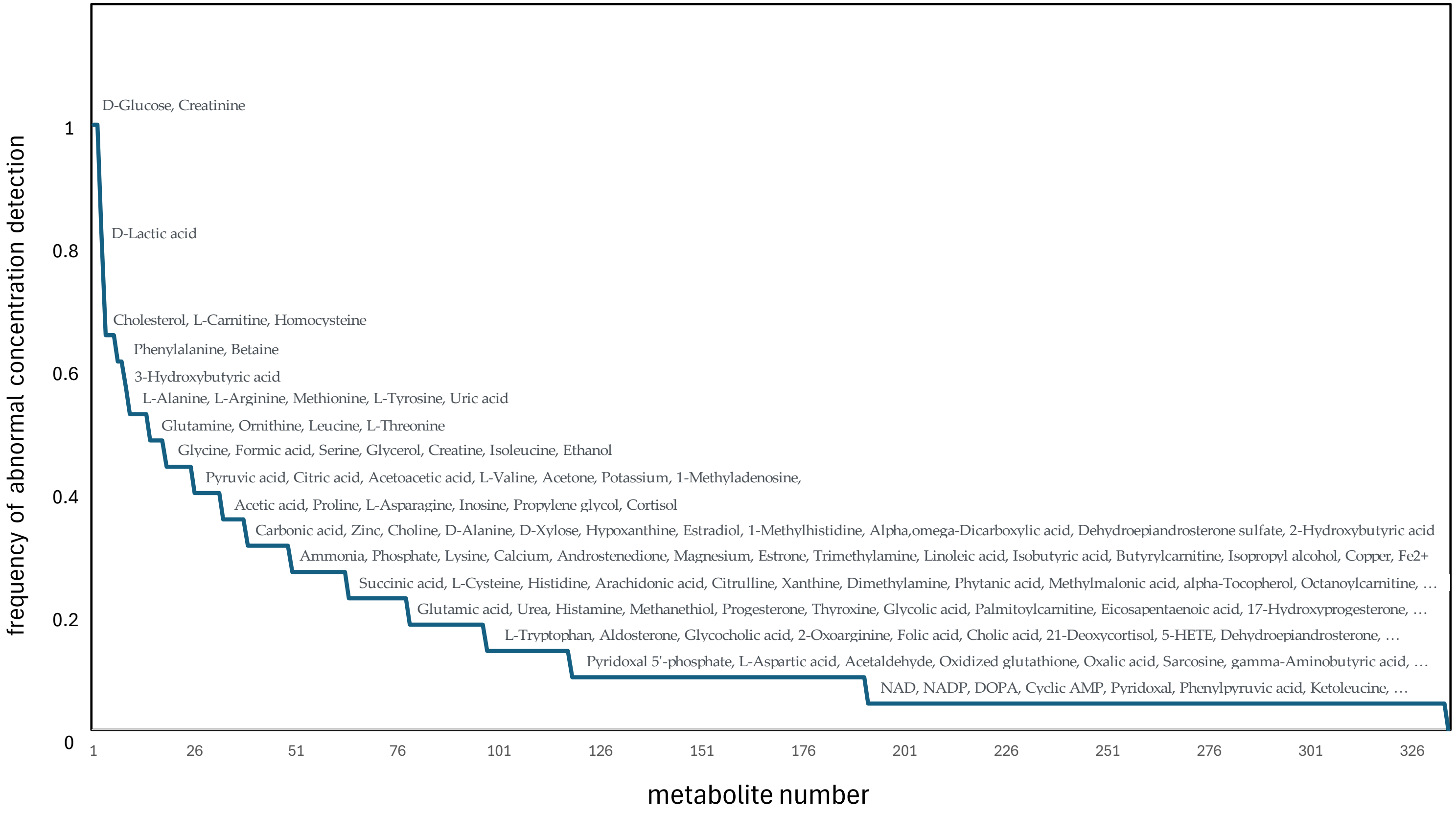

.

Figure S2. Frequency of detection of abnormal metabolite concentration in different human body conditions. Data were obtained from the Human Metabolome Database (see Table S2). The frequency is normalized to 1, which corresponds to the maximum frequency of 23 detections (demonstrated by D-glucose and creatinine).

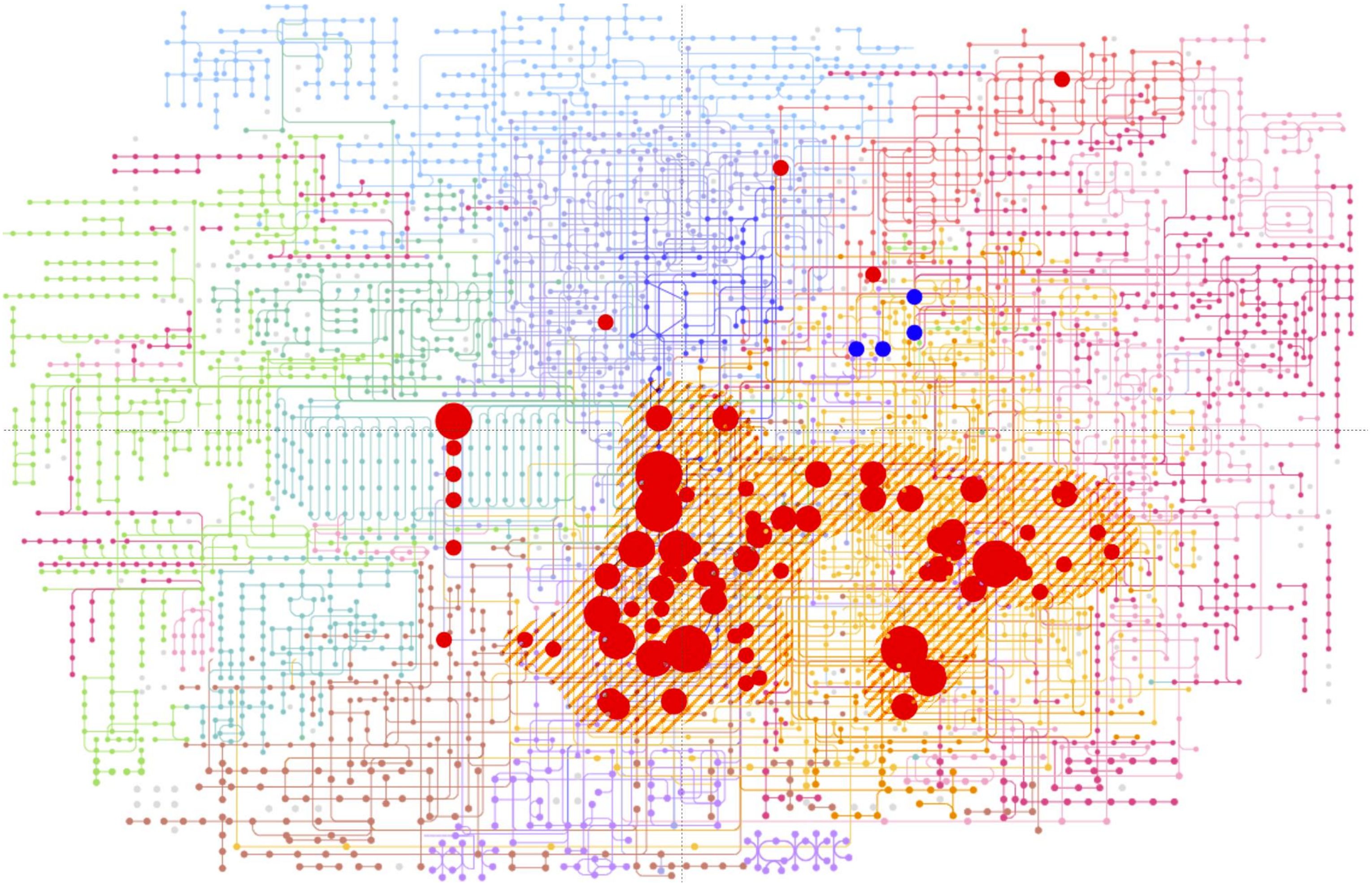

Figure S3. Projection of metapathway metabolites onto the KEGG global pathway network. Metapathway metabolites are highlighted in red circles. Blue circles refer to phenylalanine, tyrosine, and tryptophan biosynthesis pathway. The circle size shows the number of metabolite hits. The dashed area shows the main localization of the metapathway in human metabolism. The image was generated by the MetaboAnalyst program (network analysis module).
