# Supplementary material for: Aging and Pathological Conditions Similarity Revealed by Meta-Analysis of Metabolomics Studies Suggests the Existence of the Health and Age-Related Metapathway
Source: Metabolites. 2024 Nov 4;14(11):593. doi: 10.3390/metabo14110593 (PMC11597009; doi:10.3390/metabo14110593)
Supplement: Supplementary file 1 [file metabolites-14-00593-s001.zip › Supplementary Table S1.pdf]

Table S1. List of untargeted metabolomics studies on aging and identified aging-associated metabolites.

Title of study	Ref.	Cohort	Age of subjects	Sample type	Sample volume	Method of detection [1]	Instruments of detection	Number of detected compounds	Revealed age-associated metabolites [2]
Analysis of the adult human plasma metabolome.	[25]	269 healthy subjects (131 males and 138 females)	20–65 years	Blood plasma	100 µl	LC–MS, GC–MS	Surveyor HPLC with ESI+/- coupled to an LTQ MS, Thermo-Finnigan Trace DSQ fastscanning single-quadrupole MS	Over 300 compounds (~60% by LC-MS, ~40% by GC-MS)	Theobromine, Paraxanthine, Caffeine, Cotinine, Erythritol, Iminodiacetate, α-tocopherol, Pantothenate, Nicotinamide, γ-glutamyltyrosine, γ-glutamylleucine, Urate, Allantoin, Xanthine, Hypoxanthine, Inosine, Uridine, Glycerol-3-phosphate, Stearate, Palmitoleate, Oleate, Nonanedioate, Arachidonic acid, Linoleic acid, Choline, L-α-glycerophosphoryl choline, β-hydroxybutyrate, Cholesterol, Dehydroepiandrosterone sulfate (DHEA-S), Carnitine, Phenyllactate, Indolelactate, L-Kynurenine, Histamine, 3-methylhistidine, 2-hydroxybutyrate, Hydroxypyruvic acid, N-acetylvaline, cis-Aconitate, Isocitrate, α-ketoglutarate, Succinate, Malate, Orthophosphate, Glucose, Lactate, Myo-inositol, Mannose, Leucine, Isoleucine, Valine, Histidine, Lysine, Phenylalanine, Tryptophan, Threonine, Serine, Alanine, Glutamine, Glycine, Glutamate, Tyrosine, Oxoproline, Ornithine, Urea, Hippurate, p-cresolsulfate, Creatine, Thyroxine, p-hydroxyphenyllactate
Individual variability in human blood metabolites identifies age-related differences.	[26]	30 healthy male and female (15 young and 15 elderly)	29 ± 4 years (young), 81 ±7 years (elder)	Whole blood, blood plasma, and erythrocytes	200 µl	LC–MS	Paradigm MS4 HPLC system with ESI+/- coupled to an LTQ Orbitrap MS	126 metabolites	1,5-anhydroglucitol, Acetyl-carnosine, Carnosine, Ophthalmic acid, Leucine, Isoleucine, NAD, NADP, UDP-acetyl-glucosamine, Citrulline, Pantothenate, Dimethyl-guanosine, N-acetyl-arginine, N6-acetyl-lysine, Citrulline, N-acetyl-lysine, Dimethyl-guanosine, Acetyl-carnosine, Carnosine, UDP-acetylglucosamine, Ophthalmic acid
Global metabolic profiling to model biological processes of aging in twins.	[27]	Cross-sectional cohort of 268 healthy subjects (including 125 twin pairs)	6 months–82 years	Blood plasma	100 µl	LC–MS	HILIC and RPLC with HESI-II+/- coupled to an Thermo Q Exactive plus MS	770 metabolites	Galactosyl-hydroxylysine, Proline-hydroxyproline, Androsterone glucuronide, Phenol sulfate, PC(35:3), Dehydroisoandrosterone sulfate, 4-Androsten-3beta,17beta-diol disulfate, Theophylline, Trigonelline, 5-α-androstane-3-α-17-α-diol monosulfate, Quinic acid, Glucosylgalactosyl hydroxylysine, Piperine, Decanoylcarnitine, Deoxycholic acid 3-glucuronide, Erythronic acid, Threonic acid, N2,N2-dimethylguanosine, Acetylcarnosine, L-α-glutamyl-L-lisine, 1-methyluric acid, PE(40:7), Ethylglucuronide, 2-piperidinone, PC(P-32:1), Gamma-CEHC glucuronide, C-glycosyl-tryptophan, Gamma-glutamyl-ε-lysine, Pregnanolone sulfate, 5-α-pregnan-3-β-20-β-diol monosulfate, 16alpha-Hydroxy DHEA 3-sulfate, α-N-phenylacetyl-L-glutamine, Phenyl-pyruvic acid, L- cystine, Arabitol, Xylitol, L-phenylalanine, Sn-glecero-3-phosphoethanolamine, Eicosapentaenoic acid, Decenedicarboxylic acid, 2-ketoglutaramic acid, Pregnanediol-3-glucuronide, N-(1-Deoxy-1-fructosyl)tyrosine, Allopregnanolone, D-threitol, Dodecanedicarboxylic acid, L-tryptophan, Dihydro-3-coumaric acid, 2-hydroxy-ethane sulfonate, Androsterone sulfate, D-glucuronic acid, 1-methyl-histidine, N-acetyl-glutamine, Traumatic acid, Deoxycorticosterone

Longitudinal plasma metabolomics of aging and sex.	[28]	1,212 WRAP (Wisconsin Registry for Alzheimer's Prevention) subjects with 2,344 longitudinal fasting plasma samples	Every two years from middle-aged adults for 10 years	Blood plasma	100 µl	LC–MS	Waters Acquity UPLC (HILIC and RPLC) with HESI-II+/- coupled to an Orbitrap MS	1,097 metabolites	N-acetylalanine, Hydroxyasparagine, N-acetylthreonine, N-acetylserine, 1-methyl-4-imidazoleacetate, N-Acetyl-1-methylhistidine, 1-ribosyl-imidazoleacetate, 3-methylglutarylcarnitine, 5-(galactosylhydroxy)-L-lysine, Methionine sulfone, 5-methylthioadenosine, 4-acetamidobutanoate, C-glycosyltryptophan, Vanillylmandelate, Vanillactate, N2,N5-Diacetylornithine, Erythronate, Ribonate, Arabonate, Ribulonate, Xylulonate, Gulonate, Quinolinate, alpha-CEHC glucuronide, alpha-CEHC sulfate, γ-tocopherol, β-tocopherol, Citrate, Aconitate, Epiandrosterone sulfate, 5α-androstan-3α, 17β-diol monosulfate, 5α-androstan-3 β, 17β-diol disulfate, 5α-androstan-3 β, 17β -diol monosulfate, Androstenediol (3β,17β) disulfate, Androstenediol (3α,17α) monosulfate, Androstenediol (3β,17β) monosulfate, Myristoylcarnitine, Oleoylcarnitine, 3-Hydroxybutyrylcarnitin, Adipoylcarnitine, Palmitoleoylcarnitine, Pimeloylcarnitine, 3-Methyladipoylcarnitine, Eicosenoylcarnitine, Octadecenedioylcarnitin, 3-carboxy-4-methyl-5-propyl-2-furanpropanoate, 3-hydroxy-3-methylglutarate, Trimethylamine N-oxide, Docosahexaenoate, Pregnenolone sulfate, 21-hydroxypregnenolone disulfate, 5α-pregnan-3β,20β-diol monosulfate, 5α-pregnan-3β,20α-diol monosulfate, 5α-pregnan-3α,20β-diol disulfate, 5α-pregnan-3β,20β-diol disulfate, N1-methylinosine, 3-aminoisobutyrate, Pseudouridine, 5,6-dihydrouridine, Phenylacetylglutamine, Phenylacetylglutamine sulfate, Perfluorooctanesulfonic acid, Gluconate
Metabolomic markers reveal novel pathways of ageing and early development in human populations.	[29]	6055 twins (1052 fasting serum samples and 5003 fasting plasma samples)	17–85 years	Blood plasma, blood serum	n/a	LC–MS	Waters Acquity UPLC with ESI+/- coupled to an LTQ MS	280 metabolites	Aspartate, Creatine, Creatinine, Glutamate, Serine, Threitol, Phosphate, Octanoylcarnitine, 4-androsten-3beta,17beta-dioldmonosulfate, 17beta-diol disulfate, Eicosapentaenoate, 10-heptadecenoate, Dihomo-linoleate, Myristoleate, Palmitoyl sphingomyelin, Urate, Erythritol, 1,7-dimethylurate
Large-Scale metabolomics: Predicting biological age using 10,133 routine untargeted LC–MS measurements.	[30]	~10,000 toxicologic routine blood measurements	15 -90 years	Whole blood	300 µl	LC-MS	bbCID UPLC (RPLC) with ESI+ coupled to an HR-TOF-MS	12,686 features	Cortisol, Indole-3-methylacetate, Indole-3-aldehyde, Benzoyl ecgonine, Serotonin, Kynurenate, Cyclo(leu-pro), Benzoic acid, Myristoyl-carnitine, Decanoylcarnitine, Octanoylcarnitine

[1] LC, liquid chromatography; GC, gas chromatography; MS, mass spectrometry; HPLC, high performance liquid chromatography; UPLC, ultra performance liquid chromatography; ESI, electrospray ionization source; HESI-II, heated electrospray ionization source; '+/-', positive/negative mode of detection; HILIC, hydrophilic liquid chromatography; RPLC, reverse-phase liquid chromatography; bbCID, broadband collision induced dissociation; HR-TOF-MS, high-resolution time-of-flight mass spectrometry.

[2] Metabolites that did not participate in MSEA due to they were not perceived by the MetaboAnalyst are underlined.
