# Supplementary material for: Aging and Pathological Conditions Similarity Revealed by Meta-Analysis of Metabolomics Studies Suggests the Existence of the Health and Age-Related Metapathway
Source: Metabolites. 2024 Nov 4;14(11):593. doi: 10.3390/metabo14110593 (PMC11597009; doi:10.3390/metabo14110593)
Supplement: Supplementary file 1 [file metabolites-14-00593-s001.zip › Supplementary Table S2.pdf]

**Table S2.** List of metabolites with abnormal concentrations and associated conditions.

| Conditions | Metabolite Name |
|---|---|
| 11-beta-hydroxylase deficiency | Vaccenic acid; 21-Deoxycortisol |
| 21-hydroxylase deficiency | Sodium; Dehydroepiandrosterone; 17-Hydroxyprogesterone; Creatinine; Androstenedione; Potassium; D-Glucose; 21-Deoxycortisol |
| 27-hydroxylase deficiency | Phosphorus; Vitamin D3; alpha-Tocopherol; Phytanic acid; Cholesterol; Calcium |
| 3-Hydroxy-3-Methylglutaryl-CoA Lyase Deficiency | L-Carnitine; Ammonia |
| 3-Hydroxyacyl-CoA dehydrogenase deficiency (SCHAD) | 3-Hydroxybutyric acid; D-Glucose; Ammonia |
| 3-methyl-crotonyl-glycinuria | D-Glucose; Ammonia |
| 3-methylglutaconic aciduria type II, X-linked | Pyruvic acid |
| Abetalipoproteinemia | 13-cis Retinol; Trihexosylceramide (d18:1/12:0); Ganglioside GM3 (d18:1/12:0); Tetrahexosylceramide (d18:1/12:0); alpha-Tocopherol; LacCer(d18:1/12:0); GlcCer(d18:1/12:0); Cholesterol |
| Aceruloplasminemia | Fe2+; Copper |
| ACTH deficiency, isolated | Cortisol |
| Active tobacco user | Nicotine |
| Acute arsenic poisoning | Arsenic |
| Acute ethanol intoxication | D-Lactic acid |
| Acute Infectious Pneumonia | Sulfite |
| Acute mercury poisoning | Mercury |
| Acute myelogenous leukemia (AML) | Cholesterol; D-Glucose |
| Acute seizures | L-Cystine; Citrulline |
| Addison's disease | Epinephrine |
| Adenosine kinase deficiency | Methionine |
| Adrenal hyperplasia, congenital, due to 17-alpha-hydroxylase deficiency | Dehydroepiandrosterone sulfate; Deoxycorticosterone; Estradiol; Cortisol |

| | |
|---|---|
| Adrenal hyperplasia, congenital, due to 3-beta-hydroxysteroid dehydrogenase 2 deficiency | |
| Adrenomyeloneuropathy | Erucic acid |
| Adult-type citrullinemia | Ammonia |
| After alcohol intake | (R)-Salsolinol |
| After tomato drink intake | alpha-Carotene |
| AIDS | L-Cysteine |
| Alcohol abuse | |
| Alcohol intoxication | Isopropyl alcohol; Ethanol; Acetone; Methanol |
| Alcoholism | Methanol; Acetaldehyde |
| Aldehyde dehydrogenase deficiency | Acetaldehyde |
| Alkaptonuria | Homogentisic acid; Homogentisic acid |
| Alpha-Aminoadipic aciduria | Aminoadipic acid |
| Alpha-Methylacyl-CoA racemase deficiency | Phytanic acid |
| Alzheimer's disease | Nickel; Thallium; Dehydroepiandrosterone sulfate; L-alpha-Aminobutyric acid; Methylmalonic acid; 4-Hydroxyproline; Magnesium; Cobalt; L-Tryptophan; Ornithine |
| Amyotrophic lateral sclerosis | 2-Amino-4-mercaptobutyric acid |
| Anephrism | Vitamin D3; Ergocalciferol; Calcidiol |
| Apolipoprotein C-II deficiency | Cholesterol |
| Argininemia | L-Arginine |
| Argininosuccinic aciduria | Citrulline |
| Arteriosclerosis | Taurocyamine |
| Asthma | 5-HETE |
| Athyroic patients | Iodotyrosine |
| Atrophic gastritis | |
| Autosomal dominant polycystic kidney disease (ADPKD) | 13-HODE |
| Bacterial infections (assorted) | Phenylalanine |
| Bartter Syndrome, Type 4A, Neonatal, with Sensorineural Deafness | Aldosterone; Calcidiol; Carbonic acid; Creatinine; Potassium; Calcium; Phosphate |
| Bilateral testicular adrenal rest tumors | 21-Deoxycortisol |
| Biliary atresia | Hyaluronic acid |
| Biliary cirrhosis | Ursodeoxycholic acid; Lithocholic acid; Chenodeoxycholic acid; Cholic acid |
| Bladder infections | 2-Pyrrolidinone |

| | |
|---|---|
| Branched-chain Keto Acid Dehydrogenase Kinase Deficiency | Glycolic acid; Hydroxypropionic acid; Isoleucine; Ketoleucine; L-Valine; Leucine |
| Breast cancer | 16b-Hydroxyestrone; N1-Acetylspermidine |
| Breast cancer | N8-Acetylspermidine; 1,3-Diaminopropane |
| Cachexia | Uric acid |
| Canavan disease | Cytarabine; Pseudouridine; Monodehydroascorbate; Creatinine; Xanthine; Uric acid; Uridine; Orotic acid; Inosine; Hypoxanthine; Thymidine; Oxidized glutathione; Uracil |
| Cancer | 13-cis Retinol; alpha-Tocopherol |
| Cancer - assorted types | |
| Carbamoyl Phosphate Synthetase Deficiency | Glutamine |
| Cardiac arrest | Prostaglandin F2a |
| Cardiogenic shock | Inosine |
| Cardiopulmonary resuscitation | 6-Keto-prostaglandin F1a |
| Celiac disease | Lactulose; Propionylcarnitine; Palmitoylcarnitine; Butyrylcarnitine; Octanoylcarnitine; Glycocholic acid |
| Cerebral creatine deficiency syndrome 2 | Creatinine; Guanidoacetic acid; Uric acid |
| Cerebrocortical Degeneration | |
| Cerebrotendinous xanthomatosis (CTX) | Lathosterol |
| Cervical cancer | 1-Methyladenosine |
| Cholangioma cancer | 1-Methyladenosine |
| Cholesteryl ester storage disease | Cholesterol |
| Chronic arsenic poisoning | Arsenic |
| Chronic mercury (vapor) poisoning | Mercury |
| Chronic pancreatitis | |
| Chronic progressive external ophthalmoplegia and Kearns-Sayre syndrome | D-Lactic acid |
| Chronic Renal Failure | Allantoin |
| Chronic renal failure | Guanidine; Melanin; 2-Amino-4-mercaptobutyric acid; Guanidinosuccinic acid; Methylguanidine; Creatinine; Betaine; Guanidoacetic acid; L-Arginine; Zinc |
| Cirrhosis | Cholesterol sulfate; Guanidine; p-Octopamine; 2-Oxoarginine; 7alpha-Hydroxycholesterol; Guanidinosuccinic acid; 1-Methylnicotinamide; 2-Hydroxyphenethylamine; Homo-L-arginine; Linoleic acid; Lathosterol; 4-Guanidinobutanoic acid; Propylene glycol; Tyramine; |

| | |
|---|---|
| | Methanethiol; Methanethiol; Creatine; 2,3-Butanediol; Urea; L-Aspartic acid |
| Citrullinemia type II, adult-onset | Citrulline; Ammonia |
| Classicle Refsum's disease | Phytanic acid |
| Cobalamin deficiency | Beta-Leucine; Methylmalonic acid; L-Cystathionine |
| Cobalamin deficiency | Methylmalonic acid |
| Cobalamin malabsorption | Cob(I)alamin |
| Colon cancer | 1-Methyladenosine |
| Colorectal, breast, or neuroendocrine cancer | alpha-Carotene; alpha-Tocopherol |
| Combined malonic and methylmalonic aciduria | alpha,omega-Dicarboxylic acid; Methylmalonic acid |
| Continuous ambulatory peritoneal dialysis (CAPD) | 2-Amino-4-mercaptobutyric acid; Betaine; Histamine; Zinc |
| Coronary artery disease | Lipoxin A4 |
| Coronary heart disease | Hydrogen peroxide |
| Cresol poisoning | |
| Crohn's disease | D-Xylose |
| Cronh's ileitis | |
| Cruetzfeldt-Jakob disease | 2-Amino-4-mercaptobutyric acid |
| Cryptococcus meningitis | Uric acid |
| Cushing's syndrome | Androstenedione |
| Cystic fibrosis | Ursodeoxycholic acid; Deoxycholic acid; Lithocholic acid; Chenodeoxycholic acid; Cholic acid |
| Cystinosis | Cholesterol |
| Cytochrome C oxidase deficiency | Mannitol |
| D-2-Hydroxyglutaric aciduria | 2-Hydroxyglutarate; D-2-Hydroxyglutaric acid |
| Dementia (Alzheimer's and non-Alzheimer's) | 2-Amino-4-mercaptobutyric acid; L-Cysteine |
| Dengue fever | Histidine; Phenylalanine; L-Alanine |
| Depersonalization syndrome | Corticosterone; Cortisone |
| Depression | Docosahexaenoic acid; Eicosapentaenoic acid; alpha-Linolenic acid |
| Dermal fibroproliferative disorder (hypertrophic scarring) | 1-Methylhistamine |
| D-Glyceric acidemia | Glycine |
| Diabetes | 8-Hydroxyguanine; Chromium; 1,5-Anhydrosorbitol; 1,5-Anhydrosorbitol; scyllo-Inositol; Estriol; Dodecanedioic acid; 3-Hydroxybutyric acid; D-Tagatose; Pyruvaldehyde; |

| | |
|---|---|
| | Hyaluronic acid; Uric acid; D-Lactic acid; Acetoacetic acid; Glycerol; 1-Butanol; (S)-3-Hydroxyisobutyric acid |
| Diabetes and Deafness, Maternally Inherited | D-Glucose |
| Diabetes mellitus | D-Glucose |
| Diabetic ketoacidosis | Carbonic acid; 3-Hydroxybutyric acid |
| Diabetic patients | 3-Hydroxymethylglutaric acid |
| Digeorge Syndrome | Calcium |
| Dihydropyrimidine dehydrogenase (DPD) deficiency | beta-Alanine |
| Dimethyl sulfide poisoning | |
| Dimethylglycinuria | 2-Amino-4-mercaptobutyric acid; Dimethylglycine; Cob(I)alamin; Creatinine; Betaine; Folic acid; Sarcosine; Urea |
| Diverticular disease | |
| D-Lactic Acidosis and Short Bowel Syndrome | D-Lactic acid |
| Dopamine Beta-Hydroxylase Deficiency | Dopamine; Norepinephrine |
| Down syndrome pregnancy | 2-Hydroxybutyric acid; alpha,omega-Dicarboxylic acid; Isobutyric acid; Isopropyl alcohol; 1-Methylhistidine; 3-Hydroxybutyric acid; Creatinine; Betaine; Propylene glycol; Dimethylamine; Ethanol; L-Carnitine; Creatine; D-Lactic acid; Acetone; L-Threonine; L-Valine; Acetoacetic acid; Citric acid; L-Asparagine; Proline; D-Alanine; Leucine; Glycerol; Choline; L-Tyrosine; Phenylalanine; Ornithine; Methionine; Serine; Glutamine; L-Arginine; Formic acid; Succinic acid; L-Alanine; Glycine; Acetic acid; D-Glucose; Pyruvic acid |
| Drunk driver | Ethanol |
| Early preeclampsia | 2-Hydroxybutyric acid; alpha,omega-Dicarboxylic acid; Isobutyric acid; Isopropyl alcohol; 1-Methylhistidine; 1-Methylhistidine; 3-Hydroxybutyric acid; Creatinine; Betaine; Propylene glycol; Trimethylamine; Dimethylamine; Ethanol; Isoleucine; L-Carnitine; Creatine; D-Lactic acid; Acetone; L-Threonine; L-Valine; Acetoacetic acid; Citric acid; L-Asparagine; Proline; D-Alanine; Leucine; Glycerol; Choline; L-Tyrosine; Phenylalanine; Ornithine; Methionine; Serine; Glutamine; L-Arginine; Formic acid; Succinic acid; L-Alanine; Glycine; Acetic acid; D-Glucose; Pyruvic acid |
| Eczema | Leukotriene E4 |

| | |
|---|---|
| Epilepsy | Isoleucine; Isoleucine; Citrulline; L-Threonine; L-Valine; L-Asparagine; Histidine; Leucine; L-Tyrosine; Phenylalanine; L-Tryptophan; Methionine; Glutamine; Glutamine; L-Aspartic acid; L-Alanine; Glutamic acid |
| Erythropoietic protoporphyria | Fe2+ |
| Essential hypertension | Docosahexaenoic acid; Eicosapentaenoic acid; alpha-Linolenic acid; Asymmetric dimethylarginine; Linoleic acid; Arachidonic acid |
| Ethylene Glycol Poisoning | Glycolic acid |
| Eucalyptol exposure | |
| Fabry disease | Cer(d18:1/12:0) |
| Familial lipoprotein lipase deficiency | Cholesterol |
| Familial Mediterranean Fever | Etiocholanolone |
| Familial partial lipodystrophy | Creatinine; Cholesterol; D-Glucose |
| Fanconi syndrome | Carbonic acid; Uric acid; Potassium; Phosphate |
| Fatal hydrogen sulfide poisoning | |
| Fatally injured drivers | |
| Folate Deficiency | L-Cystathionine; Folic acid |
| Formic acid intoxication | Formic acid |
| Friedreich's ataxia | Thiamine |
| Gaba-transaminase deficiency | gamma-Aminobutyric acid |
| Galactosemia | Galactitol |
| Gallstone disease | |
| Gamma-glutamyltransferase deficiency | D-Glutamic acid |
| Gestational diabetes mellitus (GDM) | Linoleic acid; Oleic acid; Arachidonic acid |
| Gitelman syndrome | Carbonic acid; Magnesium; Potassium |
| Glucocorticoid resistance | Aldosterone; Cortisol; Potassium |
| Glutathione synthetase deficiency | Pyroglutamic acid |
| Glutathionuria | (gamma-Glutamylcysteine)n-glycine |
| Glycerol intolerance syndrome | Phosphorus |
| Growth hormone deficiency | D-Glucose |
| Hawkinsinuria | Carbonic acid |
| Head trauma | Epinephrine |

| | |
|---|---|
| Heart failure | Digoxin; Aldosterone; Isoleucine; Taurine; L-Threonine; Leucine; Methionine; Serine; L-Arginine; L-Alanine; Glutamic acid |
| Heart failure with preserved ejection fraction | Bovinocidin; LysoPC(18:1/0:0); Propionylcarnitine; Palmitoylcarnitine; Butyrylcarnitine; Octanoylcarnitine; Creatinine; Betaine; Trimethylamine; L-Carnitine; L-Carnitine; L-Threonine; Citric acid; PC(32:0); L-Asparagine; Histidine; D-Alanine; Glycerol; Phenylalanine; L-Arginine; Formic acid; L-Alanine; D-Glucose |
| Heart failure with reduced ejection fraction | 2-Hydroxybutyric acid; Bovinocidin; LysoPC(18:1/0:0); LysoPC(18:1/0:0); Propionylcarnitine; Butyrylcarnitine; 3-Hydroxybutyric acid; Creatinine; Betaine; Trimethylamine; L-Carnitine; L-Carnitine; Creatine; Acetoacetic acid; PC(32:0); Glycerol; Choline; L-Arginine; Formic acid; Acetic acid |
| Heart Transplant | alpha,omega-Dicarboxylic acid; Isobutyric acid; Methylmalonic acid; Isopropyl alcohol; 3-Hydroxybutyric acid; Creatinine; Betaine; Propylene glycol; Ethanol; Isoleucine; Xanthine; L-Carnitine; Creatine; Hypoxanthine; D-Lactic acid; Acetone; L-Threonine; L-Valine; Acetoacetic acid; Citric acid; L-Asparagine; Proline; alpha-Ketoisovaleric acid; Histidine; Methanol; Leucine; Glycerol; Choline; Urea; L-Tyrosine; Phenylalanine; Ornithine; Methionine; Glutamine; Formic acid; Lysine; L-Alanine; Glycine; Acetic acid; D-Glucose; Glutamic acid; Pyruvic acid |
| Heat stress | Norepinephrine |
| Hemochromatosis, type 1 | Fe2+ |
| Hemodialysis | 13-cis Retinol; 2-Amino-4-mercaptobutyric acid; Glycolic acid; Xanthurenic acid; 4-Hydroxyproline; Betaine; Histamine; Oxalic acid; Proline; Zinc; Phosphate; Glycolic acid; Oxalic acid |
| Hemolytic uremic syndrome | Trihexosylceramide (d18:1/12:0) |
| Hepatic encephalopathy | Isovaleraldehyde; gamma-Aminobutyric acid |
| Hepatobiliary Disease | Glycocholic acid |
| Hepatocellular cancer | 1-Methyladenosine |
| Hirsutism | Dehydroepiandrosterone sulfate |

| | |
|---|---|
| HIV and diarrhea | D-Xylose |
| Homocystinuria | 2-Amino-4-mercaptobutyric acid |
| Homozygous sickle cell disease | Cyanocobalamin; Pyridoxine |
| Hydrogen sulfide fatal poisoning | Thiosulfuric acid |
| Hyper beta-alaninemia | beta-Alanine |
| Hyperammonemia | |
| Hypercholesterolemia | Cholesterol sulfate; 24-Hydroxycholesterol; CE(16:0); Cholesterol |
| Hyperlipidaemia | D-Glucose |
| Hyperlipidemia | Cholesterol |
| Hyperornithinemia with gyrate atrophy | Ornithine |
| Hyperoxalemia | Monodehydroascorbate; Creatinine; Citric acid |
| Hypertension | 20-Hydroxyeicosatetraenoic acid; (R)-Salsolinol; Docosahexaenoic acid; Eicosapentaenoic acid; alpha-Linolenic acid; p-Synephrine; p-Octopamine; Linoleic acid; Arachidonic acid; Zinc |
| Hyperthyroidism | 2-Oxoarginine; 3,5-Diiodo-L-tyrosine |
| Hypobetalipoproteinemia | Trihexosylceramide (d18:1/12:0); Ganglioside GM3 (d18:1/12:0); Tetrahexosylceramide (d18:1/12:0); LacCer(d18:1/12:0); GlcCer(d18:1/12:0) |
| Hypophosphatasia, infantile | Pyridoxal 5'-phosphate; Phosphate |
| Hypophosphatemia | Phosphate |
| Hypothyroidism | 2-Oxoarginine; Thyroxine |
| Idiopathic intracranial hypertension | Retinyl ester |
| Idiopathic oro-facial pain | Gentisic acid; 2-Pyrocatechuic acid |
| Ileocysoiplasty | |
| Impaired glucose tolerance | Uric acid |
| Infantile Refsum disease | Phytanic acid |
| Insulin-dependent diabetes mellitus(IDDM) | D-Lactic acid |
| Intrahepatic biliary hypoplasia | Glycocholic acid |
| Invasive candidiasis | |
| Irritable bowel syndrome (IBS) | |
| Ischemic heart disease | Thromboxane B2 |
| Isovaleric acidemia | Adrenic acid; Eicosadienoic acid; Docosapentaenoic acid (22n-3); Vaccenic acid; Palmitoleic acid; Nervonic acid; Erucic |

| | |
|---|---|
| | acid; Docosahexaenoic acid; Eicosapentaenoic acid; alpha-Linolenic acid; gamma-Linolenic acid; Dihomo-gamma-linolenic acid; Linoleic acid; Creatinine; Oleic acid; Arachidonic acid |
| Kidney disease | Phenylacetic acid; Imidazolone; 1-Methylguanosine; Asymmetric dimethylarginine; 7,8-Dihydrobiopterin; 1-Methyladenosine; L-alpha-Aminobutyric acid; 1-Methylhistidine; Trimethylamine N-oxide; Dimethylglycine; Trimethylamine; Dimethylamine; Ethanol; Histamine; Inosine; Nitrate; Acetone; D-Xylose; Zinc |
| L-2-hydroxyglutaric aciduria | L-2-Hydroxyglutaric acid; 2-Hydroxyglutarate |
| Late-onset preeclampsia | 2-Hydroxybutyric acid; Isopropyl alcohol; 1-Methylhistidine; 3-Hydroxybutyric acid; Creatinine; Betaine; Propylene glycol; Trimethylamine; Dimethylamine; Ethanol; L-Carnitine; Creatine; D-Lactic acid; Acetone; L-Threonine; L-Valine; Acetoacetic acid; Citric acid; L-Asparagine; Proline; D-Alanine; Leucine; Glycerol; L-Tyrosine; Phenylalanine; Ornithine; Methionine; Serine; Glutamine; L-Arginine; Formic acid; L-Alanine; Glycine; Acetic acid; D-Glucose; Pyruvic acid |
| Lead induced anemia | Lead |
| Leber Optic Atrophy and Dystonia | D-Lactic acid |
| Lecithin:cholesterol Acyltransferase Deficiency | Fe2+; Cholesterol |
| Lesch-Nyhan syndrome | Creatinine; Xanthine; Uric acid; Uridine; Hypoxanthine |
| Lesch-Nyhan syndrome | Hypoxanthine |
| Leukemia | 1-Methyladenosine |
| Lipid peroxidation | Bovinic acid |
| Lipodystrophy | Cholesterol; D-Glucose |
| Lipodystrophy, Congenital Generalized | Creatinine; Cholesterol; D-Glucose |
| Lipoid Adrenal Hyperplasia | Dehydroepiandrosterone sulfate; Dehydroepiandrosterone; 17-Hydroxyprogesterone; Androstenedione |
| Liver disease (encephalopathic) | Methanethiol |
| Liver disease (non-encephalopathic) | Methanethiol |
| Liver disease with gastrointestinal bleeding | Heparan sulfate |
| Lysinuric protein intolerance | Ornithine; L-Arginine; Lysine |
| Macular degeneration | Cadmium |

| | |
|---|---|
| Major affective disorder | Cortisol |
| Major trauma | Inosine |
| Malaria | Theobromine; Nitrite |
| Malignant melanoma or sarcoma | |
| Maple syrup urine disease (MSUD) | 3-Methyl-2-oxopentanoate |
| Maturity onset diabetes of the young, type 2 | D-Glucose |
| Meckels diverticulum | |
| Menstrual cycle | Estradiol |
| Menstrual cycle (follicular phase) | Estrone |
| Menstrual cycle (luteal phase) | Estrone |
| Menstrual cycle (midcycle) | Estrone |
| Methamphetamine (MAP) psychosis | Normetanephrine |
| Methanol poisoning | Formic acid |
| Methylenetetrahydrofolate reductase deficiency | 2-Amino-4-mercaptobutyric acid; DL-Homocystine; Methionine |
| Methylmalonic acidemia and homocystinuria | DL-Homocystine |
| Mild lead poisoning (delayed reaction times, poor concentration) | Lead |
| Minor electrocardiongraphic abnormalities and/or arrhythmia with negative coronary arteriograms | |
| Mucopolysaccharidosis IVA | Keratan |
| Multiple Sclerosis | Chromium |
| Multiple sclerosis | Manganese; Nickel; Lithium; Thallium; Fe2+; Strontium; Barium; Lead; Vanadium; Aluminum; Silicon; Neopterin; 2-Amino-4-mercaptobutyric acid; Cadmium; Tungsten; Mercury; Uric acid; Magnesium; Cobalt; Molybdenum; L-Cysteine; Calcium; Copper; Zinc |
| Multiple sclerosis | Ubiquinone-1 |
| Myocardial infarction | Tetrahydrobiopterin; L-Tyrosine; Phenylalanine |
| Myoclonic epilepsy and ragged red fiber disease | D-Lactic acid |
| Myopathic carnitine deficiency | L-Carnitine |
| Myopathy with lactic acidosis, hereditary | D-Lactic acid; D-Lactic acid; D-Lactic acid; Pyruvic acid |
| N-Acetylglutamate synthetase deficiency | |
| Nephrotic syndrome | Histamine |
| Neurodegenerative diseases | |
| Obesity | Estrone sulfate; GlcCer(d18:1/12:0); 3-Hydroxybutyric acid; alpha-Carotene |

| | |
|---|---|
| Occipital Horn Syndrome | Copper |
| Occluded vessels | Tungsten |
| Oculocerebrorenal Syndrome of Lowe | L-Carnitine; Potassium; Phosphate |
| Olivopontocerebellar atrophy (OPCA) | Thiamine |
| Oral submucous fibrosis (OSMF) | Copper |
| Ornithine transcarbamylase (OTC) deficiency | Ornithine |
| Osteoarthritis | Hydrogen sulfide |
| Osteoporosis | Retinyl ester |
| Ovarian cancer | 2-Methoxyestradiol; 1-Methyladenosine |
| Overweight | alpha-Carotene |
| Paget's disease (osteitis deformans) | Galactosylhydroxylysine |
| Parkinson's disease | Manganese; Nickel; Beryllium; Lithium; Thallium; Fe2+; Strontium; Barium; Lead; Vanadium; Aluminum; Silicon; 2-Amino-4-mercaptobutyric acid; 1-Phenylethylamine; Cadmium; Tungsten; Mercury; Magnesium; Cobalt; Molybdenum; Calcium; Copper; Zinc; Chromium |
| Partial lipodystrophy | Creatinine; Cholesterol; D-Glucose |
| Pellagra | NADP; NAD |
| Perillyl alcohol administration for cancer treatment | |
| Peripheral neuropathy | 2-Amino-4-mercaptobutyric acid; L-Cysteine |
| Peritoneal dialysis | |
| Permanent intestinal failure | Citrulline; L-Arginine |
| Peroxisomal biogenesis defect | Pipecolic acid |
| Phenylketonuria | Cysteinylglycine; 17-Hydroxyprogesterone |
| Pheochromocytoma | Metanephrine; Epinephrine; Norepinephrine |
| Phosphoribosylpyrophosphate Synthetase Superactivity | Creatinine; Xanthine; Uric acid; Hypoxanthine |
| Phytosterolemia | Isofucosterol; Stigmasterol; Campesterol |
| Pituitary Hormone Deficiency, Combined, 1 | Thyroxine |
| Polycystic ovary syndrome (PCOS) | Dehydroepiandrosterone sulfate; Androstenedione |
| Porphyria | Coproporphyrin III; Coproporphyrin I; Coproporphyrinogen I; Porphobilinogen |
| Postpartum depression | Pregnenolone |

| | |
|---|---|
| Pregnancy | 13-cis Retinol; Dimethyl sulfone; 2-Hydroxybutyric acid; Cobalamin; 2-Hydroxyestrone; LysoPC(18:1/0:0); alpha,omega-Dicarboxylic acid; Butyrylcarnitine; Octanoylcarnitine; Cyanocobalamin; Isobutyric acid; Isopropyl alcohol; 1-Methylhistidine; 3-Hydroxybutyric acid; Creatinine; Betaine; Propylene glycol; Trimethylamine; SM(d18:1/18:0); Dimethylamine; Ethanol; Progesterone; Isoleucine; L-Carnitine; Creatine; D-Lactic acid; D-Lactic acid; D-Glutamic acid; Acetone; L-Threonine; L-Valine; Acetoacetic acid; Citric acid; PC(32:0); L-Asparagine; Proline; D-Alanine; Leucine; Glycerol; Choline; L-Tyrosine; Phenylalanine; Ornithine; Methionine; Serine; Glutamine; L-Arginine; Formic acid; Lysine; Succinic acid; L-Alanine; Glycine; Acetic acid; D-Glucose; Pyruvic acid |
| Pregnancy with fetus having congenital heart defect | Dimethyl sulfone; 2-Hydroxybutyric acid; LysoPC(18:1/0:0); alpha,omega-Dicarboxylic acid; Propionylcarnitine; Palmitoylcarnitine; Butyrylcarnitine; Octanoylcarnitine; Isobutyric acid; 3-Hydroxybutyric acid; Creatinine; Betaine; Propylene glycol; Ethanol; Isoleucine; L-Carnitine; L-Carnitine; Creatine; D-Lactic acid; D-Glutamic acid; Acetone; L-Threonine; Acetoacetic acid; Citric acid; PC(32:0); Proline; D-Alanine; Leucine; Glycerol; Choline; L-Tyrosine; Phenylalanine; Ornithine; Methionine; Serine; Glutamine; Lysine; Succinic acid; L-Alanine; Glycine; Acetic acid; D-Glucose; Pyruvic acid |
| Pregnancy with fetuses with trisomy 18 | 2-Hydroxybutyric acid; alpha,omega-Dicarboxylic acid; Isobutyric acid; 1-Methylhistidine; 3-Hydroxybutyric acid; Creatinine; Betaine; Propylene glycol; Ethanol; Isoleucine; L-Carnitine; Creatine; D-Lactic acid; Acetone; L-Threonine; L-Valine; Acetoacetic acid; Citric acid; L-Asparagine; Proline; D-Alanine; Leucine; Glycerol; Choline; L-Tyrosine; Phenylalanine; Ornithine; Methionine; Serine; Glutamine; L-Arginine; Formic acid; Succinic acid; L-Alanine; Glycine; Acetic acid; D-Glucose; Pyruvic acid |
| Pregnene hydroxylation deficiency | Progesterone |

| | |
|---|---|
| Premenstrual dysphoric disorder | Alloepipregnanolone |
| Premenstrual dysphoric disorder (follicular stage of menstrual cycle) | Pregnenolone |
| Premenstrual dysphoric disorder (late luteal stage of menstrual cycle) | Pregnenolone |
| Premenstrual dysphoric disorder (mid luteal stage of menstrual cycle) | Pregnenolone |
| Prepartum depression | Allopregnanolone; Epipregnanolone; 5a-Pregnane-3,20-dione; Pregnenolone |
| Primary biliary cirrhosis | Desmosterol; Cholic acid; Phenylpyruvic acid; Bovinic acid |
| Primary Hypomagnesemia | Sodium; Creatinine; Chloride ion; Uric acid; Magnesium; Potassium; Cholesterol; Urea; Calcium; D-Glucose |
| Progressive dementia | DOPA |
| Progressive supranuclear palsy | 2-Amino-4-mercaptobutyric acid |
| Proprotein Convertase 1/3 Deficiency | Liothyronine; Thyroxine; Estradiol; Cortisol; D-Glucose |
| Prostate cancer | Selenomethionine; Hydrocinnamic acid |
| Protoporphyria, Erythropoietic | Coproporphyrin I; Uroporphyrin I; Protoporphyrin IX |
| Psychiatric disorder | Tetrahydrodeoxycorticosterone; Allopregnanolone; Dihydrotestosterone; 5a-Pregnane-3,20-dione; Progesterone |
| Purine nucleoside phosphorylase (PNP) deficiency | |
| Purine nucleoside phosphorylase deficiency | Inosine |
| Quetiapine poisoning | |
| Refractory localization-related epilepsy (RLE) | L-Cystine; Serine; Lysine; Glycine |
| Refsum's Disease | Phytanic acid |
| Renal tubular acidosis, distal, RTA type 1 | Carbonic acid; Chloride ion; Potassium |
| Renovascular disease | 20-Hydroxyeicosatetraenoic acid |
| Reye's Syndrome | Aflatoxin B1 |
| Rheumatoid arthritis | 5-HETE; Folic acid; Hydrogen sulfide; Pyridoxal 5'-phosphate |
| Rhinitis | 5-HETE |
| Ribose-5-phosphate isomerase deficiency | L-Arabitol; Ribitol; Mannitol; L-Xylulose; L-Arabinose; myo-Inositol; D-Ribose |
| Sarcosinemia | Sarcosine; Serine; Glycine |
| Schizophrenia | Malondialdehyde; 5-Hydroxyindoleacetic acid; Vanylglycol; 13,14-Dihydro-15-keto-PGE2; (gamma-Glutamylcysteine)n-glycine; Linoleic acid; 3-Hydroxybutyric acid; N-Acetylserotonin; Serotonin; D-Serine; Cortisol; Betaine; gamma-Glutamylcysteine; Isoleucine; Kynurenine; Creatine; Gluconic acid; D-Lactic acid; Taurine; Arachidonic acid; D- |

| | |
|---|---|
| | Glutamic acid; L-Threonine; L-Valine; Histidine; Oxidized glutathione; Leucine; L-Tyrosine; L-Tyrosine; Phenylalanine; L-Tryptophan; Methionine; Serine; Glutamine; Lysine; L-Alanine; Glutamic acid; Homovanillic acid |
| Seizures, sensorineural deafness, ataxia, mental retardation, and electrolyte imbalance (SESAMES) | |
| Sepsis | Atorvastatin |
| Septic shock | Inosine; Tetrahydrobiopterin; Adenosine |
| Severe coronary artery disease | Estradiol; Estrone |
| Severe lead poisoning | Lead |
| Short bowel syndrome | beta-Sitosterol; D-Ornithine; Ammonia |
| Sickle cell anemia | 2-Amino-4-mercaptobutyric acid |
| Sickle cell disease-SS (SCD-SS) | Pyridoxal |
| Sildenafil-induced headache | Cyclic GMP; Cyclic AMP |
| Small intestinal bacterial overgrowth | D-Xylose |
| Small intestinal malabsorption, HIV, & cryptosporidiosis | D-Xylose |
| Small intestinal malabsorption, HIV, & diarrhea | D-Xylose |
| Small intestinal malabsorption, scleroderma, & bacterial overgrowth | D-Xylose |
| Smith-Lemli-Opitz syndrome | 7-Dehydrocholesterol; Mevalonic acid |
| Smoking | Nicotine; Tetrahydrobiopterin; Carbon monoxide |
| Solid tumors | Inosine; Hypoxanthine; Adenine |
| Stomach cancer | 1-Methyladenosine |
| Stroke | 2-Amino-4-mercaptobutyric acid; 3-Hydroxyanthranilic acid; L-Cysteine |
| Subarachnoid haemorrhage | Epinephrine |
| Subarachnoid hemorrhage | Norepinephrine |
| Thymidine phosphorylase deficiency | Deoxyuridine |
| Tic disorder | Cortisol |
| Transaldolase deficiency | Dihydroxyacetone phosphate; D-Sedoheptulose 7-phosphate; D-Ribulose 5-phosphate |
| Trauma | Nitrite |
| Type I diabetes | Lutein; alpha-Carotene |
| Uremia | 1-Methylguanosine; 1-Methyladenosine; Taurocyamine |
| Very long-chain acyl-CoA dehydrogenase deficiency (vLCAD) | Palmitoylcarnitine; Butyrylcarnitine; Octanoylcarnitine |
| Viral Infection | L-Tyrosine |

| | |
|---|---|
| Viral infections (assorted) | Phenylalanine |
| Vitamin B12 deficiency | Cyanocobalamin |
| Vitamin E deficiency | alpha-Tocopherol |
| Wilson's disease | Copper |
| Wolfram syndrome 1 | Sodium |
| Xanthinuria type 1 | Xanthine; Uric acid; Inosine; Hypoxanthine |
| X-linked ichthyosis | Dehydroepiandrosterone sulfate; Dihydrotestosterone; Estrone sulfate; Estradiol; Estrone; Androstenedione |
| Zellweger syndrome | Pipecolic acid |
