# Supplementary material for: Aging and Pathological Conditions Similarity Revealed by Meta-Analysis of Metabolomics Studies Suggests the Existence of the Health and Age-Related Metapathway
Source: Metabolites. 2024 Nov 4;14(11):593. doi: 10.3390/metabo14110593 (PMC11597009; doi:10.3390/metabo14110593)
Supplement: Supplementary file 1 [file metabolites-14-00593-s001.zip › Supplementary Table S3.pdf]

Table S3. Metabolite-metabolite interaction network parameters for nodes (metabolites).

Metabolite Name	Degree	Betweenness
L-Glutamic acid	57	234.89
Oxoglutaric acid	53	88.12
Adenosine triphosphate	51	179.78
Pyruvic acid	50	82.26
NADP	46	82.82
Carbon dioxide	46	80.72
NADH	44	108.28
Oxygen	40	52.65
L-Aspartic acid	39	110.55
Coenzyme A	39	52.55
Glycine	39	42.36
L-Glutamine	38	59.8
Citric acid	38	41.51
Glyoxylic acid	37	36.02
Succinic acid	36	47.81
Glycerol	35	36.64
L-Alanine	35	23.99
Oxalacetic acid	33	20
L-Leucine	33	13.75
L-Phenylalanine	32	18.28
L-Threonine	31	12.12
Hydrogen peroxide	30	18.93
L-Arginine	30	16.06
L-Serine	30	10.7
Magnesium	28	29.71
L-Malic acid	27	7.92
L-Tyrosine	27	6.62
L-Valine	27	5.64
Acetyl-CoA	26	13.03
Succinyl-CoA	25	17.64
Ornithine	25	14.98
L-Isoleucine	25	4.39
Hydroxypyruvic acid	24	9.35
Propionyl-CoA	24	5.09
Acetoacetyl-CoA	23	16.41
Glycolic acid	22	10.25
cis-Aconitic acid	22	5.19
FAD	21	9.82
2-Ketobutyric acid	21	2.14
Citrulline	20	5.06
Alpha-ketoisovaleric acid	20	4.4
Carbamoyl phosphate	18	6.13
Gamma-Aminobutyric acid	18	5.67
Isocitric acid	18	2.44
2-Phospho-D-glyceric acid	17	5.18
Urea	17	3.29
Phenylpyruvic acid	17	3.03

L-Asparagine	15	0.65
4-Hydroxyphenylpyruvic acid	14	3.03
Succinic acid semialdehyde	14	1.03
N-Acetylornithine	13	1.33
Glyceric acid	12	0.93
Butyric acid	11	5.04
Argininosuccinic acid	11	0.37
3-Hydroxy-3-methylglutaryl-CoA	10	2.59
Tartronate semialdehyde	10	0.56
Glucosamine 6-phosphate	10	0.44
L-Asparagine	15	0.65
Ureidosuccinic acid	9	0.36
Guanosine monophosphate	8	3.68
2-Keto-glutaramic acid	8	1.7
N-Acetylglutamic acid	8	0.18
scyllo-Inositol	7	4.9
Crotonoyl-CoA	7	1.13
4-Hydroxy-2-oxoglutaric acid	7	0
5-Phosphoribosylamine	6	0.43
Phosphoglycolic acid	6	0.12
(R)-3-Hydroxybutyric acid	5	0
N-Acetyl-L-aspartic acid	4	69
Adenylsuccinic acid	4	0.43
N-Acetylaspartylglutamic acid	1	0
